# In Vivo Quantification of Cardiac-Pulsatility-Induced Motion Before and After Double-Branched Endovascular Aortic Arch Repair

**DOI:** 10.1177/15266028221086474

**Published:** 2022-03-30

**Authors:** Jaimy A. Simmering, Steven J. G. Leeuwerke, Robbert Meerwaldt, Clark J. Zeebregts, Cornelis H. Slump, Robert H. Geelkerken

**Affiliations:** 1 Division of Vascular Surgery, Department of Surgery, Medisch Spectrum Twente, Enschede, The Netherlands; 2 Multi-Modality Medical Imaging Group, Technical Medical Centre, University of Twente, Enschede, The Netherlands; 3 Division of Vascular Surgery, Department of Surgery, University Medical Center Groningen, University of Groningen, Groningen, The Netherlands; 4 Robotics and Mechatronics Group, Technical Medical Centre, University of Twente, Enschede, The Netherlands

**Keywords:** branched endovascular aorta repair, thoracic endovascular aortic repair, ECG-gated computed tomography, aorta dynamics, stent-graft dynamics, aortic arch aneurysm

## Abstract

The Relay^®^Branch stent-graft (Terumo Aortic, Sunrise, FL, USA) offers a custom-made endovascular solution for complex aortic arch pathologies. In this technical note, a modified electrocardiography (ECG)-gated computed tomography (CT)-based algorithm was applied to quantify cardiac-pulsatility-induced changes of the aortic arch geometry and motion before and after double-branched endovascular repair (bTEVAR) of an aortic arch aneurysm. This software algorithm has the potential to provide novel and clinically relevant insights in the influence of bTEVAR on aortic anatomy, arterial compliance, and stent-graft dynamics.

## Introduction

The Relay® Branch stent-graft (Terumo Aortic, Sunrise, FL, USA) offers a custom-made endovascular solution for complex aortic arch pathologies. The main body is deployed inside the aortic arch and contains a window with endo-tunnels for branches to the brachiocephalic trunk (BCT) and left common carotid artery (LCCA). Placement of the endoprosthesis is usually preceded by a LCCA-left subclavian artery (LSA) bypass. Although branched thoracic endovascular repair (bTEVAR) has shown promising results regarding early mortality of approximately 6%, thromboembolic events remain a particular concern with an incidence up to 15%.^1–3^ Also, cerebral events have been reported to occur during follow-up after bTEVAR at 2^3^ and 95^4^ months, though mid- and long-term follow-up are often lacking. These clinical observations urged to gain biomechanical relevant insights in the influence of bTEVAR on aortic anatomy, arterial compliance, and stent-graft dynamics. However, most studies are limited to *ex vivo* benchtop fatigue testing or clinical outcomes, so *in vivo* visualization and quantification of stent-graft dynamics may help to better understand the mechanisms behind treatment success and failure.^5,6^ The purpose of this technical note was to describe a modified electrocardiography (ECG)-gated computed tomography (CT)-based algorithm to quantify geometry and cardiac-pulsatility-induced stent-graft motions of the RelayBranch. This can be useful in gaining insights in potential mechanical causes of clinical events, device durability, and ultimately give direction to improvement of the stent-graft designs.

### Case Description

**Figure 1.**
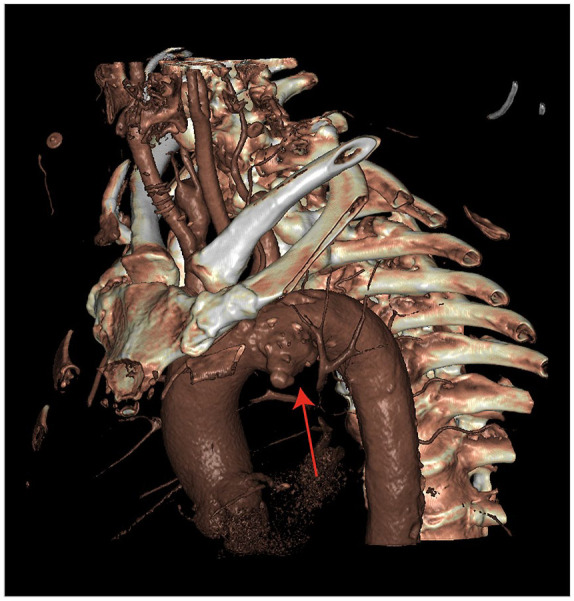
Three-dimensional (3D) rendering of part of the preoperative computed tomography (CT) volume, showing the saccular aneurysm in the aortic arch (red arrow).

A 62-year-old male was referred to our clinic with an increasing, asymptomatic saccular aortic arch aneurysm of 55 mm (Figure 1), which was discovered and followed up after myocardial infarction 3 years earlier. His medical history further mentioned sarcoidosis, hypertension, obstructive sleep apnea, diabetes mellitus, transient ischemic attack (TIA), and cerebral infarction 38 and 20 months before aneurysm treatment, respectively. He used dual antiplatelet therapy (acetylsalicylic acid 80 mg once a day and clopidogrel 75 mg once a day). After multidisciplinary consultation, bTEVAR was advised based on detailed aortic arch morphology CT-examination. In addition to the aneurysm, preoperative CT revealed a high mural thrombus load in the descending aorta. Owing to the comorbidities, open surgical repair was considered too high risk. The patient successfully underwent a 6 mm Dacron LCCA-LSA bypass with in the same session subsequent bTEVAR with the RelayBranch stent-graft (Table 1). Completion angiography confirmed proper positioning of the stent-graft without signs of endoleak or complications. Intraoperatively, the patient was heparinized and dual-antiplatelet therapy was resumed postoperatively. During the postoperative course, the patient was reintubated on the first postoperative day due to a hematoma localized at the LCCA-LSA bypass incision, which was evacuated. Discharge was on the 11th postoperative day without any further complications.

**Table 1.** The Native Aortic Arch and RelayBranch Stent-Graft Specifications.

	Size (mm)
Arterial section	
Aortic arch	
Ascending aorta diameter	43
Descending aorta diameter^a^	31
Brachiocephalic artery	
Proximal diameter	15
Distal diameter	16
Left common carotid artery	
Proximal diameter	9
Distal diameter	11
Stent-graft section	
Main device	
Proximal diameter	48
Distal diameter	40
Length	220
Brachiocephalic stent	
Proximal diameter	13
Distal diameter	18
Length	100
Left common carotid stent	
Proximal diameter	13
Distal diameter	10
Length	110

a Measured at the level of vertebrae T8.

During the next 22 months, the patient developed three separate events of embolic thrombosis of the right axillary, brachial, and radial artery. The first two events were managed by open surgical thrombectomy. After the first event, dual antiplatelet therapy was substituted for a direct oral anticoagulant (DOAC; apixaban 5 mg twice a day). After the second thromboembolic event, carbasalate calcium (100 mg once a day) was added to the DOAC. After the third event, angiography revealed an irregularity in the axillar artery, which was overstented using a 12-mm Blueflow self-expandable stent (Plus Medica, Düsseldorf, Germany). During the same period, the patient experienced recurrent TIAs, with transient visual blurriness, diplopia, and dizziness. Cerebral magnetic resonance imaging (MRI) showed recent right-sided subcortical ischemia and multiple bilateral areas with ischemia of older date. No signs of thrombosis, dissection, kinking, or geometric deformations of the endograft, potentially related to increased thrombogenicity, were found on any of eight follow-up CT scans by radiologist examination. Discussions with a second-opinion neurologist, vascular internist and vascular teams of other endovascular dedicated centers revealed no explanation for the cerebral embolisms. Eventually, the patient died of hemorrhagic stroke 22 months after bTEVAR. Autopsy was not performed in accordance with the wishes of the family.

## Methods

The Institutional Ethical Review Board of the Institution approved this study according to the Dutch Act on Medical Scientific Research involving Human Beings (WMO). Data were stored and analyzed anonymously.

### Device Description

The RelayBranch stent-graft is a custom made solution for aortic arch pathology, based on the Relay Non-Bare Stent (NBS) platform (Terumo Aortic, Sunrise, FL, USA). Therefore, the stent-graft and delivery device properties of the main body are similar to the NBS. The RelayBranch differs with the addition of a window in the proximal part of the graft with two tunnels equipped with barbs, to facilitate fixation of the branches to the BCT and LCCA.^7^

### Image Acquisition

The patient underwent ECG-gated CT imaging preoperatively, 11 days postoperatively and 5.5 months postoperatively. The scans were performed on a Somatom® Definition Flash CT scanner (Siemens Healthcare, Erlangen, Germany) during breath-hold, with the patient in supine, feetfirst position, and administration of 80 mL intravenous contrast agent (Visipaque 320 mg I/mL, GE Healthcare, Chicago, IL, USA) administered at 4 mL/second. The CT rotation time was 0.3 s, with collimation 64 × 0.6 mm, slice thickness 1 mm, slice increment 0.5 mm, reconstruction matrix 512 × 512 pixels, convolution kernel i36f, pixel size ranging 0.6 × 0.6 − 0.8 × 0.8 mm, tube voltage 120 kV with automated tube current modulation and automated pitch factor based on the heart rate. Retrospective gating allowed reconstruction of 10 equally sized CT volumes, representing 10 phases of the cardiac cycle from 0 to 90% of the R–R interval. A previously established image registration algorithm^5,8^ was used to obtain a phase-averaged CT volume and 10 deformation fields. The deformation fields can be used to translate voxels of the phase-averaged CT volume to the corresponding locations in the 10 cardiac phases. This allowed a single measurement to be translated to each cardiac phase and thereby diminish the observer dependence.

### Cardiac-Pulsatility-Induced Motion

**Figure 2.**
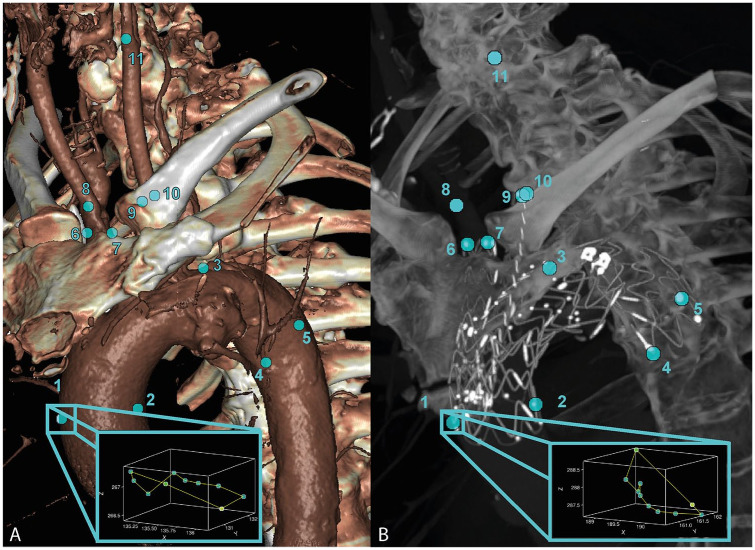
The selected points for motion amplitude calculations for the preoperative (A) and postoperative (B) electrocardiogram (ECG)-gated computed tomography (CT) scans. Points 1 and 2: ventral/dorsal ascending aorta and first stent ring, respectively; point 3: top of the aortic arch; points 4 and 5: ventral/dorsal descending aorta and last stent ring, respectively; points 6 and 7: (end of the) brachiocephalic trunk (BCT) (stent); 8 on the BCT bifurcation; points 9 and 10: (end of the) left common carotid artery (LCCA) (stent); 11 on the LCCA bifurcation. The blue-bordered boxes show the path traveled by point 1 during the cardiac cycle in the two scans.

Quantification of the cardiac-pulsatility-induced motion of the native vessel and RelayBranch was done by manual selection of points in the phase-averaged CT volume: the ventral and dorsal ascending aorta (1&2), the top of the aortic arch (3), the ventral and dorsal descending aorta (4&5), the end of the BCT (6&7), the BCT bifurcation (8), the LCCA (9&10), and the LCCA bifurcation (11) (Figure 2). The motion amplitudes, that is, pulsatility, of selected points in x —(lateral), y —(ventral-dorsal), and z —(caudal-cranial) directions were calculated by adding the deformation fields to the point coordinates as described and validated by Koenrades et al^5^ with an error of ≤0.3 mm. The pathlength of each selected point was calculated as the sum of the distances between the point locations in subsequent phases.

### Geometrical Parameters

The geometrical parameters included length, tortuosity index (TI), and curvature of the BCT, LCCA, and aortic arch, and were calculated over centerlines obtained with Aquarius Intuition (TeraRecon, Inc, Foster City, CA, USA). The centerlines were sampled at 1 mm by interpolation. A Savitzky–Golay filter of polynomial order 4 and window length 33, was applied to the centerlines to suppress noise and inaccuracies and to obtain a differentiable curve up to the second order, as necessary for curvature calculations. The ends of the stent were marked in all scans to derive the geometrical parameters of the stented part of the arteries. Anatomical landmarks derived from the first postoperative CT were used to find these locations preoperatively, that is, the to be stented centerline segment. Length was defined as the sum of distances between the centerline coordinates and TI as this length divided by the straight distance from start to endpoint of the centerline or centerline segment. Curvature is an accurate characterization of the curviness for each centerline point which has been described previously^9^ and can be calculated numerically according to equation 1:

(1) $$\kappa =\frac{\sqrt{{\left(z^{\prime \prime }y^{\prime }-y^{\prime \prime }z^{\prime }\right)}^{2}+{\left(x^{\prime \prime }z^{\prime }-z^{\prime \prime }x^{\prime }\right)}^{2}+{\left(y^{\prime \prime }x^{\prime }-x^{\prime \prime }y^{\prime }\right)}^{2}}}{{\left({x^{\prime }}^{2}+{y^{\prime }}^{2}+{z^{\prime }}^{2}\right)}^{\frac{3}{2}}},$$

in which (x,y,z) are the Cartesian coordinates of the centerline and ′ and ″ are the first and second derivatives of the coordinates, respectively. The cardiac-pulsatility-induced changes in the geometrical parameters were calculated on the centerlines of the individual cardiac positions, after translation of the phase-averaged centerline to the phases using the deformation matrices.^5^ For curvature, the difference between the minimal and maximal value during the cycle for each point was calculated, resulting in a value for each centerline point. The average and maximum of these values were defined as respectively the average and maximal change in curvature during the cardiac cycle.

### Diameter Change

**Figure 3.**
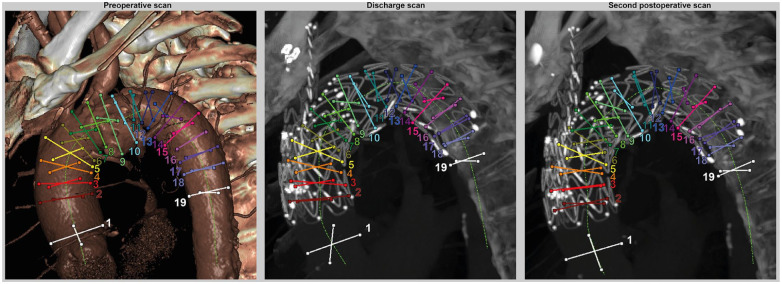
The calculated diameters at the three scan moments: preoperative, before discharge and at 5.5 months postoperative, that is, second postoperative scan. Two diameters were calculated at each 1 cm level of the centerline (bright green), forming a diameter pair. Each diameter pair is shown in a different color with a corresponding number. The white diameter pairs indicate the native aorta diameters upstream and downstream of the main body in the aortic arch.

The 3mensio Vascular workstation 10.1 (Pie Medical Imaging BV, Maastricht, The Netherlands) was used to obtain a 3D segmentation of the aortic arch lumen in the phase-averaged CT volume with a smoothing factor of 5. Diameters of this segmentation were calculated in the planes perpendicular to the centerline with steps of 1 cm along the centerline of the stented aortic arch. For these centerline locations, two lines perpendicular to the centerline tangent were calculated. The locations where these perpendicular lines crossed on the 3D aortic arch segmentation were marked for the diameter calculations: the two marked points for each perpendicular line were used to calculate one diameter, resulting in two perpendicular diameters at each level along the centerline. In addition, diameters of the native aorta upstream and downstream of the stented aortic section were calculated. Diameter change, that is, pulsatile expansion, of the aortic arch was calculated as the difference between maximal and minimal diameter over the cardiac cycle by translating the marked points to each cardiac phase. The two diameters per centerline level were averaged into a mean diameter value per centerline level of 1 cm, that is, a diameter pair (Figure 3).

## Results

### Cardiac-Pulsatility-Induced Motion

**Figure 4.**
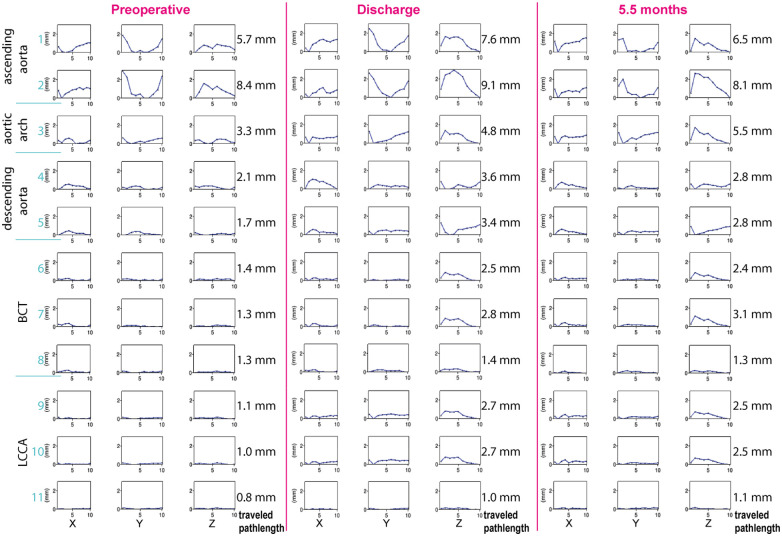
The motion amplitudes in x —(lateral), y —(ventral-dorsal), and z —(caudal-cranial) direction and the corresponding pathlengths at the different scan moments (preoperative, discharge and 5.5 months postoperative) in which the motion pattern of each coordinate (X, Y, or Z) is shown in the columns for each point (1–9 as specified in Figure 1). The sum of individual distances between each phase for each point is specified as the traveled pathlength for each point.

Figure 4 shows the motion amplitudes in the *X*-, *Y*-, and *Z*-direction and traveled pathlength for each selected point at the different scan moments. The motion in the *Z*-direction and pathlength increased after bTEVAR, especially for the branches (ie, BCT 6&7; LCCA 9&10). For these points, the maximal *Z*-amplitude increased from 0.22 to 0.89 mm, 0.20 to 0.93 mm, 0.19 to 0.80 mm, and 0.21 to 0.35 mm, respectively. In addition, after bTEVAR, the difference between the pathlength of the point on the most distal stent ring of the branch stent and the first bifurcation of the branches increased by 1.18 mm in the BCT and 1.68 mm in the LCCA, while the first-bifurcation-pathlengths changed <0.3 mm. The cardiac-pulsatility-induced stent-graft motion is also shown in a video, supplied as supplementary online material (Video 1).

### Geometrical Parameters

The geometrical parameters of the (to be) stented parts of the BCT, LCCA and aortic arch centerlines at the different scan moments are presented in Table 2.

**Table 2.** The Geometrical Parameters (Length, TI, and Curvature) of the (to be) Stented Parts of the BCT, LCCA, and Aortic Arch at the Different CT Scan Moments.

	BCT			LCCA			Aortic arch		
	Preoperative CT	Discharge CT	Second postoperative CT	Preoperative CT	Discharge CT	Second Preoperative CT	Preoperative CT	Discharge CT	Second Preoperative CT
Length—mid cardiac cycle (mm)	85.6	90.7	90.7	106.7	110.5	113.7	176.1	173.7	167.6
Length—cardiac-pulsatility-induced change (mm)	1.1	1.4	1.4	1.5	1.6	1.8	3.3	2.4	2.3
TI—mid cardiac cycle	1.048	1.031	1.032	1.024	1.005	1.005	1.860	1.773	1.720
TI—cardiac-pulsatility-induced change	0.007	0.002	0.001	0.002	0.001	0.002	0.066	0.067	0.041
Mean curvature—mid cardiac cycle (m^−1^)	25.4	13.9	14.2	20.4	7.1	7.0	23.6	23.7	21.9
Maximal curvature—mid cardiac cycle (m^−1^)	77.0	27.5	26.9	47.3	18.6	24.1	50.3	41.1	40.0
Mean curvature—cardiac-pulsatility-induced change (m^−1^)	2.1	1.5	1.5	1.7	1.5	1.3	1.6	1.7	1.3
Maximal curvature—cardiac-pulsatility-induced change (m^−1^)	3.7	2.4	2.9	4.4	3.3	2.4	4.3	4.5	3.8

The phase-averaged, that is, static parameters, were calculated in the phase-averaged CT volume and the cardiac-pulsatility-induced changes are the differences between the largest and smallest value of the parameter during the cardiac cycle.

Abbreviations: BCT, brachiocephalic trunk; CT, computed tomography; LCCA, left common carotid artery; TI, tortuosity index.

**Figure 5.**
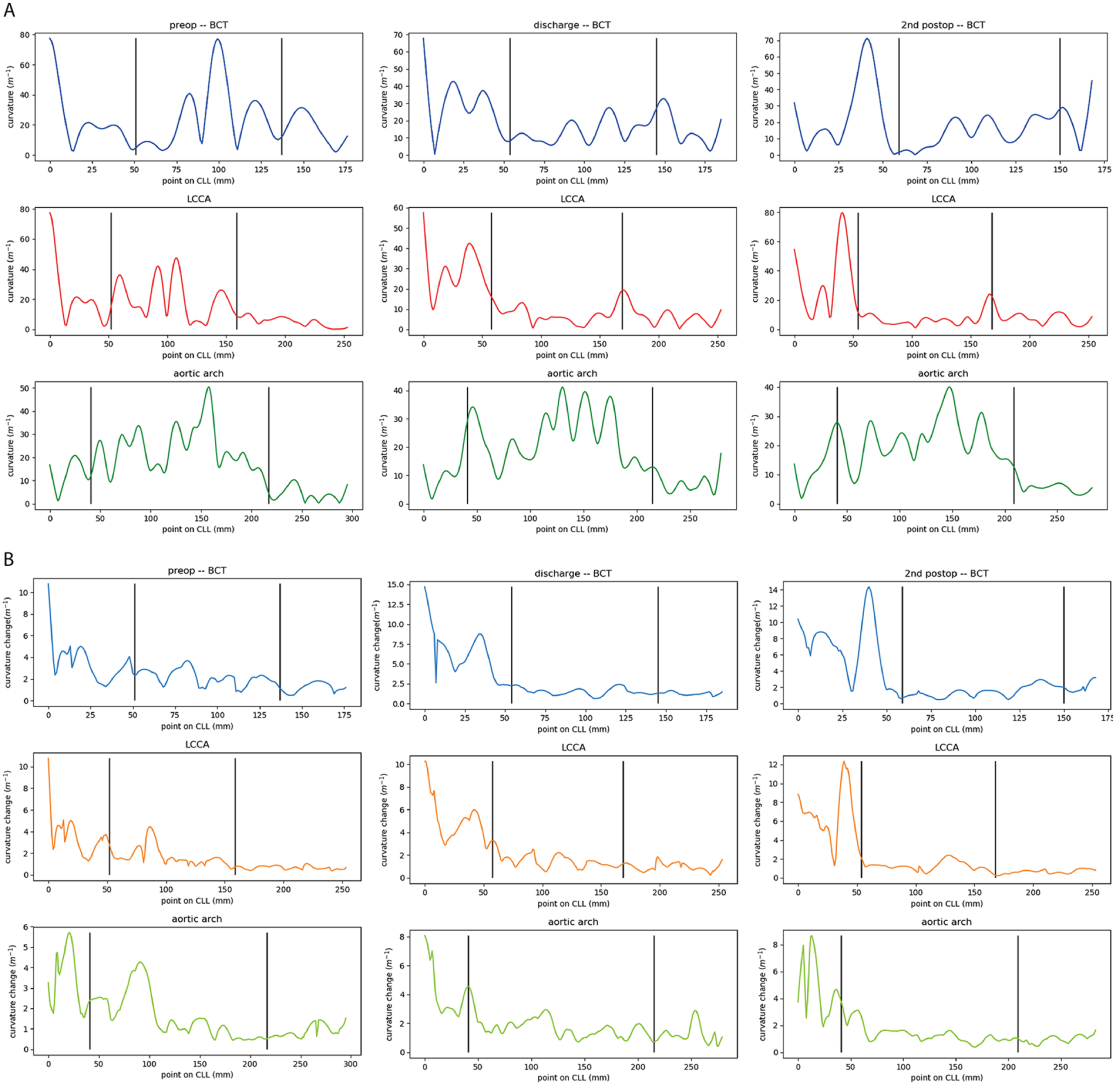
The (A) static phase-averaged curvature and (B) curvature change of the pre-operative (left), discharge (middle) and 2^nd^ postoperative (right) electrocardiogram (ECG)-gated computed tomography (CT) scan of the brachiocephalic trunk (BCT, blue), left common carotid artery (LCCA, red/orange) and aortic arch (green). The vertical black lines indicate the start and end of the to be stented part of the arteries. CLL, center lumen line.

After bTEVAR, mean and maximal curvature decreased by 11.5 and 49.5 m^−1^ respectively for the stented BCT and 13.3 m^−1^ and 28.7 m^−1^, respectively for the stented LCCA. The mean curvature of the stented aortic arch remained constant over all scan moments between 21.9 and 23.7 m^−1^, while maximal curvature showed a decrease of 9.2 m^−1^ from pre- to post-bTEVAR. Mean and maximal curvature in the phase-averaged CT volume of the stented arteries remained constant during follow-up (change < 2 m^−1^), except for the LCCA, which showed an increase in maximal curvature, location at the distal end of the stent (Figure 5).

The cardiac-pulsatility-induced mean curvature change remained constant after bTEVAR with change < 0.6 m^−1^. Furthermore, the location of the maximal curvature of the aortic arch is not the same as the location of the maximal cardiac-pulsatility-induced change in curvature during the cardiac cycle in any of the scans (Figure 5).

### Diameter Change

**Figure 6.**
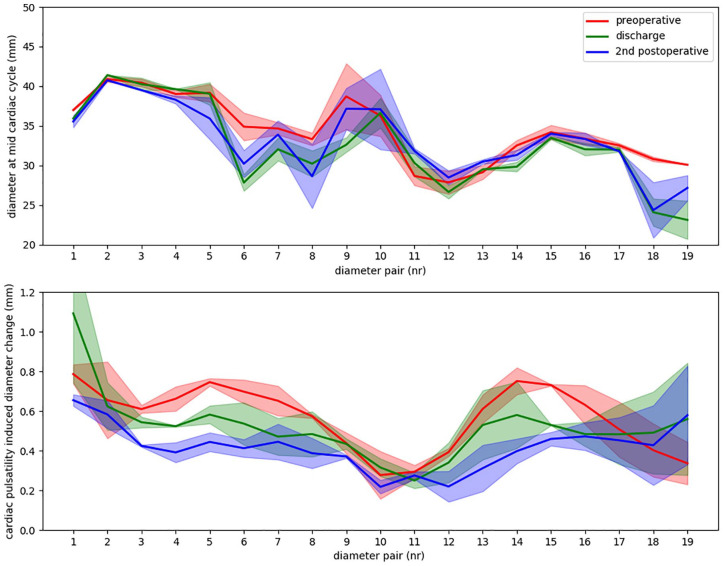
The diameters of the different scan moments (A) and the corresponding cardiac-pulsatility-induced changes in diameter (B) for the diameter pairs along the centerline, spaced at 1 cm of the centerline of the stented aortic arch and a diameter of the of the native aorta upstream (diameter pair 1) and downstream (diameter pair 19) of the stent. The curves show the mean diameter value (calculated from the diameter pair), whereas the filled areas around the curves show the spread of the 2 diameters of each diameter pair.

A decrease in phase-averaged diameter after bTEVAR can be observed in Figure 6 for the diameters at the level of the window in the device (diameter pairs 5-8). At these levels and the first levels thereafter, the segmentations are also more oval, seen as a larger spread around the mean diameters in Figure 6. Furthermore, the cardiac-pulsatility-induced diameter change was <1 mm at all scan moments, except for the first diameter (upstream native ascending aorta) at discharge. During follow-up, the diameter change of the unstented ascending aorta reduced to below the preoperative value. The smallest diameter change was at the middle of the aortic arch, at the level of the aneurysm and the first diameter thereafter (diameter pairs 10-12) at all scan moments (Figures 3 and 6).

## Discussion

This technical note shows the potential of detailed dynamic analysis of ECG-gated CT scans, comprising longitudinal and cardiac-pulsatility-induced changes in motion patterns, geometrical parameters, and diameters. The analyses of a single patient showed a remarkable increased cardiac-pulsatility-induced motion in *z*-direction and pathlength of the stented aortic arch and its branches. Interestingly, the distal ends of the branch stents (BCT and LCCA) showed this increase in *z*-direction motion and pathlength, while no change in these variables was seen at the first bifurcation of the branches. The stented aortic arch and side branches were straightened after bTEVAR (reduction in maximal curvature), probably due to the stiffness of the stent, and limited cardiac-pulsatility-induced bending of the aortic arch and side branches. Furthermore, the diameter of the aortic arch and its pulsatile expansion remained generally stable after bTEVAR, with only minor reduction in diameter change between subsequent scans (Figure 6B).

Although bTEVAR is technically feasible, thromboembolic events remain a major concern.^1,2,10^ Previous studies show that implantation of a stent-graft in the aorta increases aortic stiffness and can increase cyclic deformation in the adjacent native vessels.^11^ The increase in aortic stiffness may change flow velocities and wall shear stress both within and outside of the stent-graft, which for bTEVAR may influence cerebral hemodynamics as well.^11,12^ Furthermore, several studies have related aortic stiffness to stroke.^11,13,14^ Herman et al.^15^ assumed that the introduction of branches in the stent-graft design increases platelet activation potential, consequently increasing the risk of thromboembolic complications. Van Bakel et al.^16^ underlined with computational fluid dynamics simulations that aortic arch endograft design could have significant impact on hemodynamic performance. This may potentially affect cerebral blood flow during follow-up and the need to monitor the long-term outcomes in this cohort of patients. However, a stiffer stent does not necessarily impair the outcome because a straight artery encounters less flow disturbances^17^ and therefore could be less suspect to adverse events. Increased motion of the distal ends of the BCT and LCCA branch stents after bTEVAR was observed in this patient while the first branch bifurcations motion remained constant. Thereupon we hypothesize that this difference in motion is to be compensated in the artery between these locations. In other words, the artery between the distal end of the stent and the first carotid artery bifurcation may undergo increased deformation with every heartbeat. This vessel wall deformation may be increasing the risk of thromboembolic complications noted by others.^15^ The observed limited cardiac-pulsatility-induced change in curvature and diameters are considered to aid in device durability. However, it is postulated that this localized increased deformation in the outflow artery of the branches may induce intimal micro-damage, which may ultimately lead to the formation of microembolisms that could migrate to the brain and arms. As this hypothesis is based on the results of a single patient, it should be interpreted with care.

Recently, Suh et al.^18^ performed analysis on preoperative and postoperative ECG-gated CT scans of 11 regular, nonbranched Gore G-TAG and Cook TEVAR patients and investigated centerline parameters and diameters of the aorta as well. The maximal curvature, both phase-averaged (40-50 m^−1^ vs ca. 0.4-0.5 cm^−1^) as dynamic (3.8-4.5 m^−1^ vs ca. 0.15-0.30 cm^−1^), were of the same order as found in the patient in this study, though they saw overall lower and stronger decreasing cardiac-pulsatility-induced curvature change. A plausible explanation may be that a smaller pulse wave amplitude is experienced when placing a regular TEVAR device in the more straight descending aorta downstream of the LSA, compared to the aortic arch that we investigated. Furthermore, the diameter calculations of Suh et al. also show that the cardiac-pulsatility-induced diameter change of the ascending aorta increased post-TEVAR. However, several methodological differences between Suh et al. and this study should be noted. First, they did not perform CT volume registration to reduce user dependence and increase the signal-to-noise ratio (SNR). Second, they only investigated one postoperative ECG-gated CT at different times during follow-up. Therefore, remodeling was not taken into account while we investigated two postoperative ECG-gated CT scans, which showed remodeling continues during follow-up. Finally, they also took into account the curvature of the inner and outer curve of the aorta, though with circle fitting instead of numerical computation, while we only reviewed the centerline curvature.

Although no definitive conclusions can be drawn from our study with a single patient, the novel approach of in depth ECG-gated CT analyses may provide added advantage of broad new knowledge of bTEVAR longitudinal and dynamic behavior when applied to a larger cohort. A first step in future research would be to repeat this study in a larger cohort. In addition, it would be interesting to investigate if similar results are observed in devices with a comparable design, such as the Cook Branched Arch Endograft, and devices with a different branched-attachment design, such as the currently in trial Gore TAG Thoracic Branch Endoprosthesis and Cryolife Endospan. Still, derived from the presented results, the direction for improvement of stent-graft design could be to aim for reduction of the difference in aortic branch motion of the stented and native artery. The first step in this direction could be to reproduce these results in a larger patient cohort and investigate cardiac-pulsatility-induced motion in branched stent-graft designs that are more flexible and conformable than the currently used stents, for example, Anaconda limbs (Terumo Aortic, Sunrise, FL, USA). In addition, since multiple potential causes of thromboembolic stroke after aortic arch repair have been identified,^15^ combining these results with other *in vivo* flow studies (such as computational fluid dynamics^16^ or ultrasound particle image velocimetry),^19^ may shine more light on the cause of thromboembolic stroke following bTEVAR in the aortic arch.

## Conclusion

Quantitative detailed ECG-gated CT analysis of the aortic arch before and after endovascular repair of an aortic arch aneurysm with a RelayBranch device is technically feasible and may provide novel, clinically relevant insights in the influence of bTEVAR on aortic anatomy, arterial compliance, and stent-graft dynamics. Analysis of one patient showed that bTEVAR increases aortic arch and branch stiffening and increased cardiac-pulsatility-induced motion in the *Z* direction in the branch outflow arteries. When performed in a larger patient cohort, these analyses have the potential to unravel the pathophysiology behind observed complications after bTEVAR and give direction to further improve future stent-graft designs.

## References

[1] van Bakel TM de Beaufort HW Trimarchi S , et al. Status of branched endovascular aortic arch repair. Ann Cardiothorac Surg. 2018;7(3):409–416. doi:10.21037/acs.2018.03.13. PMC6094020 30155420

[2] van der Weijde E Heijmen RH van Schaik PM , et al. Total endovascular repair of the aortic arch: initial experience in the Netherlands. Ann Thorac Surg. 2020;109(6):1858–1863. doi:10.1016/j.athoracsur.2019.09.009. 31593657

[3] Chen L Dai X Wu X , et al. Ascending aorta and hemiarch replacement combined with modified triple-branched stent graft implantation for repair of acute. Ann Thorac Surg. 2017;103(2):595–601. doi:10.1016/j.athoracsur.2016.06.017. 27553503

[4] Zhang L Lu Q Zhu H , et al. Branch stent-grafting for endovascular repair of chronic aortic arch dissection. J Thorac Cardiovasc Surg. 2021;162(1):12–22. doi:10.1016/j.jtcvs.2019.10.184. 31926697

[5] Koenrades MA Struijs EM Klein A , et al. Quantitative stent graft motion in ECG gated CT by image registration and segmentation: in vitro validation and preliminary clinical results. Eur J Vasc Endovasc Surg. 2019;58(5):746–755. doi:10.1016/j.ejvs.2019.03.009. 31548160

[6] Koenrades MA Donselaar EJ van Erp MAJM , et al. Electrocardiography-gated computed tomography angiography analysis of cardiac pulsatility-induced motion and deformation after endovascular aneurysm sealing with chimney grafts. J Vasc Surg. 2020;2013. doi:10.1016/j.jvs.2020.01.064. 32249042

[7] Ferrer C Cao P. Endovascular arch replacement with a dual branched endoprosthesis. Ann Cardiothorac Surg. 2018;7(3):366–371. doi:10.21037/acs.2018.04.08. 30155415 PMC6094012

[8] Klein A Kroon D-J Hoogeveen Y , et al. Multimodal image registration by edge attraction and regularization using a B-spline grid. Med Imaging 2011 Image Process. 2011;7962:796220. doi:10.1117/12.878267

[9] Simmering JA Slump CH Geelkerken RH , et al. Geometrical change in Anaconda endograft limbs after endovascular aneurysm repair: a potential predictor for limb occlusion. Semin Vasc Surg. 2019;32(3–4):94–105. doi:10.1053/j.semvascsurg.2019.11.001. 32553125

[10] Kudo T Kuratani T Shimamura K , et al. Early and midterm results of thoracic endovascular aortic repair using a branched endograft for aortic arch pathologies: a retrospective single-center study. JTCVS Tech. 2020;4:17–25. doi:10.1016/j.xjtc.2020.09.023. 34317956 PMC8307048

[11] Nauta FJH van Bogerijen GH Trentin C , et al. Impact of thoracic endovascular aortic repair on pulsatile circumferential and longitudinal strain in patients with aneurysm. J Endovasc Ther. 2017;24(2):281–289. doi:10.1177/1526602816687086. 28102102

[12] Zhu Y Zhan W Hamady M , et al. A pilot study of aortic hemodynamics before and after thoracic endovascular repair with a double-branched endograft. Med Nov Technol Devices. 2020;4(December 2019):100027. doi:10.1016/j.medntd.2020.100027.

[13] Laurent S Katsahian S Fassot C , et al. Aortic stiffness is an independent predictor of fatal stroke in essential hypertension. Stroke. 2003;34(5):1203–1206. doi:10.1161/01.STR.0000065428.03209.64. 12677025

[14] Al-Qamari A Adeleke I Kretzer A , et al. Pulse pressure and perioperative stroke. Curr Opin Anaesthesiol. 2019;32(1):57–63. doi:10.1097/ACO.0000000000000673. 30543556 PMC6310080

[15] Herman CR Rosu C Abraham CZ. Cerebral embolic protection during endovascular arch replacement. Ann Cardiothorac Surg. 2018;7(3):397–405. doi:10.21037/acs.2018.04.09. 30155419 PMC6094019

[16] Van Bakel TM Arthurs CJ Van Herwaarden JA , et al. A computational analysis of different endograft designs for Zone 0 aortic arch repair. Eur J Cardio-Thoracic Surg. 2018;54(2):389–396. doi:10.1093/ejcts/ezy068. 29554234

[17] Hobbie RK Roth BJ , eds. Mechanics. In. Intermediate Physics for Medicine and Biology. 4th ed. Springer; 2006:1–29.

[18] Suh G Bondesson J Zhu YD , et al. Multiaxial pulsatile dynamics of the thoracic aorta and impact of thoracic endovascular repair. Eur J Radiol Open. 2021;8:100333. doi:10.1016/j.ejro.2021.100333. 33748348 PMC7957153

[19] Engelhard S Voorneveld J Vos HJ , et al. High-frame-rate contrast-enhanced US particle image velocimetry in the abdominal aorta: first human results. Radiology. 2018;289(1):119–125. doi:10.1148/radiol.2018172979. 30015586

